# Pennsylvania College, Ninth below Locust Street

**Published:** 1853-01

**Authors:** W. H. Gobrecht


### CLINICAL REPORTS.

---

*Pennsylvania College, Ninth below Locust street. Service of* Professor GILBERT.

Reported by W. H. GOBRECHT, M. D.

*Nov. 20th.* CASE XXXVIII.—*Congenital Teleangiectasy. Remarks, History of Case and Operation.*—This is an unnatural expansion in the capillary vascular system, producing, sooner or later, swellings which have their seat in the skin alone, in the subjacent areolar tissue alone, or in both, but rarely extends more deeply. It may be *congenital* or *non-congenital.*

The color of the tumor is quite red when the arterial capillary dilatation is the greatest; or it is bluish when the venous implication is in excess.

These tumors enlarge and pulsate, giving a *crawling* sensation, even to the patient himself. And after a time, when a great size has been attained, hæmorrhage occurs from the rupture of some spot in the thinned skin, which, however, closes again, this hæmorrhage recurring frequently.

The tumor is composed of enlarged and convoluted capillaries held together by loose areolar tissue, and perhaps cavities filled with blood.

The causes of Teleangiectasy are obscure; it is usually congenital, and is met with on all parts of the surface of the body, but especially about the cranium and face, the skin of which is highly vascular.

Teleangiectasy is essentially a local disease, and hence local appliances must be looked to in effecting a cure. These are varied in their character, and may be thus principally enumerated:

1. *Compression.* Successful only in very slight degrees of the disease.

2. *Destruction*, with the actual and potential cauteries, when we have subsequently an ordinary granulating and suppurating sore.

3. *By producing sufficient inflammation* in the Teleangiectasic tissue *to obliterate the vessels*, by vaccination, punctures, &c.

4. *By tying the principal supplying artery.* This is not so effectual as other methods, being, in great part, palliative in its character.

5. *By extirpation*, either with the knife, or by strangulation with the ligature. The use of the knife is not always safe, on account of the vast number of bleeding orifices which we create, from which alarming hæmorrhage will most assuredly ensue, if the tumor be large. If, however, this plan is employed, we must cut wide of the pulsating mass and in the sound tissue.

Having said thus much, the case of the patient was brought to the notice of the class. John G——, from Ireland, aged 20, has been affected since his birth with an erectile tumor of his lower lip, which, small at first, has increased with his growth, and still increases. It involves at present the skin, mucus membrane, and subjacent areolar tissue from a little to the right of the median line of the lower lip to somewhat beyond the left angle of the mouth. The gums in the immediate vicinity are deeply involved, and bleed often. The tumor is bluish red, thick, pouting, indefinitely circumscribed, and merges somewhat into the cheek above the oral angle; it also gives us the crawling pulsation, and can be emptied, in great measure, of its contents by pressure. It is stated that spontaneous hæmorrhage frequently occurs.

On carefully manipulating the tumor we can trace the main trunks of supply to the facial arteries, which vessels are found to be much enlarged. And inasmuch as the tumor itself is so extensive and involves so much important tissue, we shall give the preference, at least for the present, to the operation for the *ligation of the facial artery* upon *each side*, which is the fourth method of treatment detailed in the preceding remarks. The operation was then performed as follows:

The patient being seated, the jaws were firmly closed and the anterior edge of the masseter muscle found on the left side, whereby the exact course of the artery, and the proper position for ligation, was known. An oblique incision, one inch and a quarter in length, was now made in a direction downwards and forwards from the edge of the belly of the masseter to a little below the base of the jaw and anterior to the muscle; by these means were cut, the integument, superficial fascia and m. platysma myoides. The artery was then exposed and separated from the facial vein, which lies upon its outer side, by means of forceps whose blades were fashioned into a form similar to a curved needle, the blunt extremities of which grasped the ligature, which was passed from the outside toward the median line, and then firmly tied. The same operation was performed upon the right side, and after the conclusion of these the pulsation ceased, and the size of the tumor had much diminished.

27th. The tumor is found to be smaller, and pulsation less evident than prior to the operation. Incisions nearly closed, but ligatures still retained.

CASE XXXIX.—*Contusion of Right Arm.*—George D——, aged 50. This injury, which exists upon the outer aspect of the right arm, was caused by a box weighing 800 lbs. falling some two feet and striking the patient at the point indicated. This occurred some two weeks since, the integument being unbroken; upon the third day after, swelling commenced, which progressed, accompanied with pain and redness, until an abscess formed; this was punctured some 48 hours back, and about half a pint of pus evacuated, since when the cavity has been contracting and the quantity of discharge is diminishing. The injury inflicted by this box, similates that of a spent cannon ball. The force being applied obliquely to the part, disorganized the areolar and muscular structures beneath without any external wound; inflammation was set up within, and abscess formed to get rid of the destroyed tissues, which act as foreign bodies, besides being too extensive for absorption alone.

Mrs. L., (Case XVI) who had a finger amputated for obstinate flexion, is presented, and the stump shown to have healed perfectly.

CASE XL.—George A——, aged 18. This patient *stepped upon a needle*, which breaking, left a fragment in his foot, whilst crossing the floor, protected by his stockings alone, the usual cause of such accidents. Pain is evinced upon pressure at one point of the sole, but as this is deep, it is not deemed advisable to cut down into it, but rather to elevate the limb, poultice the foot, and await the formation of an abscess around the foreign body, with the contents of which it will be discharged.

CASE XLI.—*Nævus Maternus*, in Mary Ellen B——, aged 22 months. This is one of the forms of congenital Teleangiectasy, consisting of an enlargement of the capillary vessels in and beneath the integument. It was first noticed as presenting the appearance of a minute scratch ; since then it has enlarged gradually to its present size. which is that of a split filbert, placed upon the left ala of the nose. The proper plan of treatment, it was stated, will be to remove it without delay, which was done thus: An incision was made around the tumor, in the sound integument entirely, a straight needle was then passed beneath the base of the erectile mass from above, downward and backward, which was elevated by raising its extremities, and a surgeon's needle, carrying a double ligature, passed beneath the mass and the transfixing pin, at right angles to the latter. The two extremities of the respective cords (after the bight of the ligature had been cut) were tied firmly beneath the straight pin in the track of the integumental incision on their respective sides, the knots lying opposite each other, and the pin withdrawn. The color was now found to have changed from a bright red to a dull, bluish white. In two or three days we will find that the tumor has sloughed away, from the abstraction of all its sources of vascular and nervous supply.

24th. The tumor has become detached, and a freely granulating surface presents itself, which was touched with argent. nit. to repress an exuberant tendency which existed. The isinglass plaster was applied.

*Nov.* 24th. CASE XLII.—*Varus of right foot, third degree*, which occurred in a male child now aged 11 months. The operation for the cure of this affection, was performed during the last winter before the class, in the child's fifth week, by dividing the tendo Achillis by Hypodermatomy, and applying a steel sole, and leg bar for the outside of the limb, the method of employing which is given in the Examiner for December 1852, p. 785. The child is shown as presenting to us almost perfect relief from the deformity, but as there is a slight tendency to the inversion of the foot, a stiff and high gaiter has been constructed with whalebones in its outer side, which will retain the dorsum in its proper relation to the axis of the limb.

Case XLIII. *Reducible scrotal hernia, oblique descent.*—Danl. S——, aged 25, states that when in his 19th year, he strained himself, as he supposes; about two weeks after this time, a tumor presented itself in the right groin, which could be made to disappear on pressure. He has worn a number of trusses, differently constructed, but could never continue them for any length of time; the tumor consequently gradually increased in dimensions, and eventually descended into the scrotum. On examination, it is found that the recumbent posture, with slight manipulation, reduces the mass entirely, especially if we relax the muscles concerned by flexing the thigh of the affected side on the pelvis, and carrying the knee to the opposite limb. Hull's truss was then applied. This instrument is thought by Prof. Gilbert to be the best, inasmuch as its construction is the least complicated; it is therefore less liable to get out of order, and can be more readily repaired should it do so, besides which it answers effectually all the indications as an easy retentive appliance. Simplicity being here, as in other instances, the best test of usefulness.

Remarks on the subject of hernia in general, and the varied means of diagnosis, were also made in conjunction with the presentation of the case.

Case XLIV.—Mary G——, aged 37. *Contusion of shoulder and side*—produced by stumbling and falling upon a stairway. The patient had not fracture of the ribs, since there was no local *catch* in a long inspiration, and no crepitation. The motions of the humerus were proper. Treated with the application of volatile liniment and a bandage to the thorax and arm.

Case XLV. *Abscess beneath fascia lata of left thigh.*—Ellen C——, aged 8 years. This patient was brought to us for advice several weeks ago. She then complained of pain at the upper part of the thigh, which was tender to the touch. There being also lameness, it was supposed that there might be incipient Morbus Coxarius. The Tinct. of Iodine was prescribed as a local application. The Comp. powder of Jalap ordered as a purgative, and rest enjoined. To-day, we have a further development of the case, viz., tumor, which exhibits its true condition. We shall, however, defer any decided measures until the next Clinical day.

Dec. 1st. The left thigh presents a prominence just outside of the median line of the limb, and about 2½ inches below the groin; this gives fluctuation, though perhaps rather obscurely. If pus exists here, as we think it does, a timely opening should be made to prevent the infiltration of the tissues, which would certainly occur, beneath the firm fascia which binds them down so closely; and this puncture we should not neglect, as is often done, for fear of the great vessels, until a large collection of pus results. If the incision is made at the proper point outside of the axis of the limb, and in a line *parallel* with the great vessels, very little is risked. A puncture was accordingly made at the point indicated above, and about two ounces of watery and curdy discharge, with a few drops of blood, followed. The wound was dressed with dry lint and adhesive strips.

4th. No discharge to-day at all; adhesive strip applied. There is much less pain in the thigh.

*Nov.* 27th. Daniel D——, (Case XXXII) previously exhibited —returns with increased irritation of the lachrymal sac, resulting from the epidemic Influenza, most probably. Creasote was re-applied. Pil. cathart. comp. No. III, were ordered—and the local application of the Alum curd directed, should the irritation run into an inflammation.

Dec. 11th. This patient has improved somewhat; there is no very apparent dryness of the nose; no inflammation of the conjunctiva at the inner canthus of the eye; there is not such frequent necessity of pressure upon the sac to empty it; and but a single tear, regurgitated by pressure with the finger of the manipulator, is clear and devoid of mucus admixture. Notwithstanding, however, these signs of palliation, Prof. Gilbert states it as his experience, that in cases of this character, we cannot hope to do more than bring the affection to a stand, and to keep it there by treatment, going on as they inevitably do, if neglected, to entire closure of the duct, finally resulting in fistula lachrymalis.

*Dec.* 1st. *Congenital varus* in an infant, exhibited. This is to be operated for upon the 8th inst.

Case XLVI.—*Strabismus* in a child aged 4 years, exhibited, with remarks upon its treatment.

*Deformity of Neck from Burn*, presented, with some history of the case. To be operated for, on 4th inst.

CASE XLVII. *Severe contusion of knee joint.*—Mr. M., aged 25, about eighteen months ago fell and injured the knee joint by contusion ; from this, however, recovery soon occurred. Some fourteen months since, he fell and contused the same joint again, very severely. For this liniments were employed, and the patient confined himself to the house. Recovery, however, being delayed, medical advice was sought. As synovitis was evidently commencing, indicated by pain, swelling, heat, redness and inability to use the joint, Prof. Gilbert at first confined the patient to his bed, and applied the straight back splint, extending from near the pelvis to a point just above the heel, to prevent any motion, and employed repeated blistering about the joint, as a means of counter-irritation. These blisters were followed, upon healing, by Tinct. Iodine, pencilled over the parts. The Guano poultice was next used, and then blisters again, the leg being extended and motionless during the whole length of time; besides which a constitutional treatment, consisting of Bitartrate of Potassa and Jalap, as a revulsive, and Lugol's solution of Iodine as a sorbefacient, was instituted and persevered in.

By these means, some time in last May, seven months back, the natural size of the limb was restored, whereupon the ordinary *starched bandage* was applied, and the joint thus kept nearly motionless and extended, but the semi-recumbent posture was still enjoined. For the last two months the patient has been moving about upon the limb thus supported, perfectly free from pain, swelling, or unpleasant symptom of any description whatever, and is now attending to the ordinary duties in his shoe store in the city.

CASE XLVIII. *Contusion of hip joint.*—Salone T——, aged 14, was knocked off of a cotton bale, and falling, struck the left hip, about two weeks since. The case was properly diagnosed as contusion, by the practitioner who was called to attend him, and a treatment by cups and counter-irritants in the vicinity of the joint, established. As now presented, we find that the patient cannot elevate the thigh, without raising the knee by his hands, and dragging the heel along the mattress, and this with some pain, whilst there is none of the preternatural rigidity of dislocation, nor the abnormal mobility of fracture, existing, the limb being of its proper length and capable of being made to fulfil its proper motions by external aid. Severe contusion evidently has been the cause of the present disturbance of the functions of the part, and may result in synovitis, which in any, but especially in this boy's constitution (scrofulous,) would be exceedingly disastrous, falling, as the case undoubtedly would, into Morbus Coxarius. To prevent any motion, therefore, a *starched bandage* was applied in the presence of the class, and the thigh and knee, respectively, placed in a slightly flexed position, were there secured by a long splint, to be continued until the consolidation of the bandage. When this has occurred, the firm case formed by it may be cut upon the inside of the thigh, and the sound side of the body; being then padded with cotton, as required, it is to be re-secured by turns of a roller, and worn for several months, the patient being confined to the recumbent posture.

Dec. 4th. The case is progressing favorably in every respect.

*Dec.* 4th. J. S. (Case No. VII,) Cataract, is again shown to-day, the operation for absorption having been repeated about two weeks since. The lens was then effectually broken up, besides which, the posterior layer of the capsule (having been recently involved) was divided. The opening thus formed acts as a second pupil, and the rays of light are concentrated upon the retina through it; therefore the patient can see not only light, but can distinguish persons from other objects, and tell how many fingers are held before him readily, without any aid from a lens. It is very probable that the presence of this new pupil, by regulating the rays admitted, will enable the patient to read without the aid of glasses. Discharged cured.

Case XLIX.—*Deformity of neck from burn, and operation for its relief.*—Hannah J. S——, of Kensington, aged 14 years, was severely burned, when 2 years old, by her clothes being accidentally fired. Burns are apt to be more severe in children than in adults, since, whilst the latter, with due presence of mind, stand and subdue the flames by the most accessible methods, the former run wildly about for help, and increase the danger by fanning the flames, and igniting other parts of the clothing, which, but for this, would be, perhaps, untouched. The burn is, therefore, not only extended superficially, but deeply, and amounts, as in this instance, to the *third* or *fourth degree*, where the deep tissues are involved, and absolute loss of substance ensues. No doubt, had this child been somewhat older, more serious consequences still, if not death, would have ensued, from the reasons just given.

But the extent and depth of the burn are not the only difficulties which we have to encounter in the treatment and cure of such cases. No sooner are the sloughs thrown off by the process of ulceration, than very exuberant, large and flabby granulations are quickly formed, which, contracting, draw together the edges of the ulcer, like the mouth of a purse, and then skinning over, constitute a thick, uneven cicatrix, composed of what is termed *inodular tissue.* But the work is still unfinished. Interstitial absorption of this mass, if not previously set up, now commences, and thus the contractility of the tissue is again increased by these newly combined actions. As a consequence, very little new substance eventually exists in the space occupied by the burn; and that which is there becoming firm, hard and almost ligamentous, the contraction still progressing, the cicatrix is frequently raised from the substances beneath, especially if it have points of attachment, as upon either side of the flexure of joints, &c. In the case before us, the most serious portion of the burn seems to have been in the front of the neck, extending deeply; in consequence of which, all the steps in the cure, first described, have been successively established, and the great contractility of the cicatrix has approximated the lower jaw to the sternum in such a manner that all depression of the neck has vanished, the chin has been shortened and drawn backward, whilst the teeth project forward, away from the line of the arch of the upper jaw, and the head has a general inclination forward and downward, giving the patient a downcast look.

The long continued deformity has had the effect of measurably arresting the growth and expansion of the lower part of the face and jaw, and demands an operation, for the purpose of allowing the progression of proper development and the production of unrestricted powers of mastication, as well as for the comfort of the individual and the relief of the unsightly deformity.

The patient being seated, a transverse incision was made with a scalpel on the anterior part of the neck, from right to left, about 6 inches in length, dividing the adventitious structure down to the normal fascia beneath, and the head extended, whereby the chin became instantaneously prominent. At a point some 2½ inches to the *right* of the extremity of the first cut, the scalpel was now made to enter the integument, and an incision extended downward on the shoulder 7 inches, then giving a rounding cut, it was carried up parallel with and 2½ inches from the first incision, entering the transverse cut at its *right* extremity.

The flap thus formed was dissected up from the muscles beneath, rotated to the left upon its base, and placed in the gaping wound produced by the separation of the lips of the first incision. Here, after the approximation of the edges of the wound in the shoulder in a like manner, it was secured by 16 fine steel needles, No. 12, and the twisted suture, with adhesive strips between them. Strips were also placed between their projecting extremities on either side and the integument, also superficial to them. The entire number of needles inserted both in the shoulder and neck was *twenty-two*, of which only *two* were of great size; one of these being placed at the angle of the shoulder and neck cuts, and the other on the shoulder itself.

The employment of a *number* of *small needles*, is an improvement on the old method of using fewer but of larger size, inasmuch as the irritation set up by the presence of the former, is not by any means so great as with the latter.

Very little blood was lost, and, although the operation from its extent was necessarily somewhat tedious, it was borne unusually well, by one so young. Anæsthesia was resorted to during the earlier stage of the proceedings, but was not employed thereafter. Patient retained at the College for subsequent treatment.

Dec. 8th.—Since last Saturday, (4th) the patient has fallen under the effects of the Epidemic Influenza, now so prevalent, which has complicated the case in a very serious manner. Distressing cough, which has a constant tendency to disturb the union of the flap, exists, together with marked febrile symptoms of no favorable kind. The treatment to this time has been (on the 6th,) Mass. Hydrarg, gr. vj. followed in two hours by Calcined Magnes. ℨij

On the 7th she had dark, copious and fetid evacuations, after which some relief of all the symptoms was experienced. She was then given:

℞. Liq. Potass. Citrat. f℥viij.

Ant. et Pot. Tart. gr. j.

M. S. f℥ss. every two hours.

The adhesive strips were for the first time removed on to-day, (the 8th) and re-applied. Union in about three-fourths of the lower border of the flap, being found nearly completed, ten of the needles were removed after the appliance of the plasters. Not much more than a fluid-drachm of pus has been formed up to this time. The fever has almost entirely subsided.

Dec. 18th.—Union by adhesion has been prevented, even in parts seemingly united by the first intention. The suppuration set up required daily dressings. On the 12th, all the needles were removed. The edges of the wound from which the flap was taken, were drawn about an inch asunder by the tension of the adjacent integument; and the extremity of the flap, by its own elasticity, parted to a like extent from its connection with the sound skin on the left side of the neck. By daily renewal of adhesive plaster, and the introduction of a suture at the extremity of the flap, the parts have been kept in apposition sufficiently well for favorable union by second intention, so that to-day (18th) the case may be reported as progressing very favorably towards union of all the parts involved in this very extensive and important operation.

*Dec.* 8th.—John P. (CASE XXVII,) *Tenotomy.*—The wrist is now shown perfectly extended, although much restricted in its movements by the long confinement it has experienced. As the punctures have perfectly healed, and the tendons have doubtless united, the splint is directed to be frequently removed and passive motion given.

CASE L.—*Congenital Varus of both feet, second degree,* in

George W. S——, aged 4 months. The tendo Achillis of each limb was divided by subcutaneous section in the usual manner, and the apparatus, whose description was given in Med. Exam. for Dec., 1852, (page 785, article, Adhesive Plaster in Surgery, by D. Gilbert, M. D. etc.,) applied as there directed.

Dec. 15th.—Apparatus removed and reapplied. The wounds have healed entirely, and the feet are nearly in their normal position. The progress so far is of the most encouraging character.

*Dec.* 11th.—A large *Tumor of the Neck*, in a female, was exhibited, and the history of the case given.

This person will be presented for the necessary operation hereafter.

*Dec.* 15th. Case LI.—*Malignant Tumors beneath Lower Jaw.*—Mr. G——, aged 60, states that about a year or two since a scirrhous tumor was formed in his lower lip. This was removed, and the parts healed well afterward. Within the last four months, however, tumors have formed below the jaw and near to its angle; these are now about the size of a walnut, and have firm attachments to the surrounding tissues. As the removal of these tumors is strongly contra-indicated, the patient is advised to abstain from all cauteries and other irritating appliances, lest ulceration and more speedy death should ensue than if they were left undisturbed.

The ordinary reliable alteratives, however, may be employed, as Donovan's solution, Comp. Decoct. of Sarsaparilla, &c., but at best we can only palliate and endeavor to avert death by guarding against improper appliances of every description.

Case LII.—*Polypus Nasi.*—George F——, aged 81 years, has a Polypus in his right nostril, which has been increasing for about two months. Several have been removed at two distinct sittings previously, by another physician. This, which was removed by torsion, was found to be of the mucous variety, and of moderate size. This case is worthy of notice on account of the polypus appearing, for the first time, so late in life.

